# DUSTBot: A duplex and stealthy P2P-based botnet in the Bitcoin network

**DOI:** 10.1371/journal.pone.0226594

**Published:** 2019-12-20

**Authors:** Yi Zhong, Anmin Zhou, Lei Zhang, Fan Jing, Zheng Zuo

**Affiliations:** 1 College of Cybersecurity, Sichuan University, Chengdu, Sichuan, China; 2 College of Electronics and Information Engineering, Sichuan University, Chengdu, Sichuan, China; Chongqing University of Posts and Telecommunications, CHINA

## Abstract

As the root cause of illegal cyber activities, botnets are evolving continuously over the last two decades. Current researches on botnet command and control mechanism based on blockchain network suffer from high economic cost, single point of failure, and limited scalability. In this paper, we present DUSTBot, a novel P2P botnet model based on Bitcoin transactions to prepare for new cyber threats. Specifically, a covert, duplex, and low-cost command and control (C&C) channel in the Bitcoin network is presented in our work. DUSTBot uses the Bitcoin main network as the downstream channel while using the Bitcoin testnet as the upstream channel. Furthermore, the peer list exchange algorithm based on the Ethereum block hash proposed in this paper is effective against routing table poisoning attack and P2P botnet crawling. The robustness of DUSTBot against node removal is studied through constructing the botnet with a P2P simulator. We deploy the implementation of DUSTBot on cloud platforms to test its feasibility and performance. Moreover, the stealthiness of DUSTBot and the effectiveness of the proposed peer list exchange algorithm are evaluated. The results demonstrate the feasibility, performance, stealthiness, and robustness of DUSTBot. In the end, possible countermeasures are discussed to mitigate similar threats in the future.

## 1. Introduction

With the fast development of the Internet, the number of devices accessing the network, especially the Internet-of-Things (IoT) devices grows continually. Gartner [1] forecasts that up to 20 billion network devices will be connected to the Internet by 2020. Thus, cybersecurity is becoming more and more critical. As the main cyber threat, botnet consists of a network of compromised computers (personal computer, mobile phones, or smart devices) controlled by a remote attacker (“botmaster”) [2]. Botnets may be the source of many cyber-attacks, including data exfiltration, E-mail spam, phishing, distributed denial-of-service (DDoS) attack [3], extortion [4], and cryptocurrency mining [5].

Compared to other Internet malware, the feature of command-and-control (C&C) communication makes the botnet unique. A covert and reliable C&C channel ensures the robustness of a botnet. Once the centralized C&C server is taken down, the botnet will also be shut down. Defenders usually analyze a botnet through network traffic [6,7], reverse engineering and techniques of honeypot [2,8]. After capturing the traces of a botnet from network traffic and analyze the captured bot through reverse engineering, the defenders can deploy honeypots to monitor the botnet and develop strategies to disrupt the botnet. [9,10].

Traditional C&C channels like IRC networks and HTTP-based communications may cause single-point-of-failure [11] because of centralized C&C architectures. The C&C server of a botnet is exposed once a bot is captured and reverse engineered by defenders, then the botmaster could be traced.

Additionally, the botmasters tried to construct their botnet based on some abnormal C&C channels, including darknets, social media, and cloud services. Sanatinia *et al*. [12] proposed OnionBot, a botnet model based on Tor services. Nappa *et al*. [13] proposed a novel botnet model that exploits an overlay network such as Skype to build a parasitic overlay. Pantic *et al*. [14] presented a steganographic system that demonstrates the feasibility of the social networking website Twitter as a botnet C&C center. Nagaraja *et al*. proposed Stegobot [15], which also uses Twitter for its C&C system. However, the C&C channels of these botnets are still centralized. Skype is a centralized cloud-based architecture after the Microsoft takeover in 2011 [16]. Defenders could effectively shut down these botnets cooperating with the network service providers.

Compared to centralized botnet architecture, a more robust decentralized P2P C&C architecture comes out. P2P botnets without centralized C&C servers avoid single-point-of-failure. Botnets such as Conficker [17], Nugache [18], and Storm Worm [19] have implemented different kinds of P2P architectures. However, P2P botnets may be vulnerable to routing table poisoning or Sybil attack [20]. Moreover, the bootstrap procedure of P2P bootstrap may also cause single-point-of-failure. Nugache botnet relies on a hardcoded bootstrap peer list contained 22 IP addresses.

To solve the problems mentioned above, concrete solutions to apply blockchain technology to build infrastructure for botnets are proposed. Some public blockchain networks [21] (Bitcoin [22], Ethereum [23], et al.) are ideal choices for botnet C&C communication because they are decentralized, public, anonymous, and robust.

Ali *et al*. proposed ZombieCoin [24,25], a botnet command and control (C&C) mechanism utilizing the Bitcoin network. ZombieCoin regularly indicates web server address as rendezvous points where bots can direct upstream data through the Bitcoin network, which make it vulnerable to traditional botnet takedown methods. Pirozzi [26] proposed BOTCHAIN, a fully functional and duplex botnet built upon the Bitcoin protocol. However, the scalability of BOTCHAIN is limited by the unbearable economic cost because of the transaction fee of Bitcoin. Malaika [27] proposed Botract, which deploys its C&C logic on the functions in smart contracts to the Ethereum blockchain. The botmaster sends and receives commands and keeping track of the state of bots through the functions of the smart contract. However, this proposal needs to download a full Ethereum blockchain on every single infected host. Up to April 2019, the size of full Ethereum blockchain exceeds 217GB [28]. Using Light Ethereum Client, the disk storage could be decreased to about 10MB. However, Ethereum Light client protocol is still under development [29]. UnblockableChains [30] is a POC project of a fully functional C&C infrastructure on top of the public Ethereum network. UnblockableChains provides secure communications, larger bandwidth, and less cost of data transfer. However, to set up a client of UnblockableChains on an infected host, 290 MB of disk space and 300MB of memory are required. It is much easier to be detected if an unknown program is consuming that much system resources.

Considering the challenges encountered by using blockchain technology as an infrastructure for a botnet, in order to deploy a duplex, cost-effective and large-scale botnet in the Bitcoin network, we present DUSTBot, a duplex and stealthy P2P-based botnet model utilizing the Bitcoin testnet [31] as the upstream channel. The testnet is a global platform to experiment with the Bitcoin protocol and its scripting capabilities. The reason why we choose Bitcoin testnet is that Bitcoin is the most stable cryptocurrency. The Bitcoin testnet uses a separate, distinct Bitcoin blockchain, and so-called faucets [32–34] to provide anyone with coins for free [35]. Therefore, the economic cost of upstream communication is negligible. We make the following significant contributions:

1. To overcome the weakness of current solution to apply blockchain technology to botnet communication (high economic cost, single point of failure and limited scalability), we proposed DUSTBot, a novel botnet model that uses Bitcoin network as its C&C channel. DUSTBot receives commands from the Bitcoin main network and sends data back to the Botmaster via the Bitcoin testnet.
2. We exploit a Bitcoin message to disguise a DUSTBot as a genuine Bitcoin node. The network behavior between bots is similar to genuine Bitcoin nodes, and communication data is embedded into illegitimate transactions.
3. To defend against routing table poisoning attack and P2P botnet crawling, we proposed a peer list exchange algorithm which utilizes the randomness and frequency of the latest Ethereum block hash as salt value.
4. We construct a simulated botnet with a P2P simulator to evaluate its properties and robustness. We also evaluate the effectiveness of the proposed peer list exchange algorithm. Moreover, we implement a prototype of DUSTBot with a Bitcoin API and deploy a small botnet on cloud platforms to test its feasibility and performance.

The rest of this paper is organized as follows. Section 2 states the related works in novel botnet C&C mechanisms research. Section 3 describes the methods used in this work, including the botnet architecture, the detailed C&C mechanism of DUSTBot, and the proposed peer list exchange algorithm. Section 4 presents the results of the experiments. Robustness, feasibility, performance, and stealthiness of DUSTBot are evaluated in this section. In addition, the effectiveness of the proposed peer list exchange algorithm is evaluated in this section. Section 5 and section 6 discusses countermeasures, economic cost, and robustness of the proposed C&C channel. Finally, we conclude this paper and present our future work in Section 7.

## 2. Related work

There are lots of studies on novel botnets with different C&C mechanisms to enhance the stealthiness, invulnerability and communication efficiency before botnets are evolved. We summarize several of them as follows:

Starnberger *et al*. [36] present Overbot, a botnet protocol based on Kademlia distributed hash table for stealth C&C communication. Queries generated by Overbot are similar to legitimate queries, which makes it hard to be distinguished from other queries. However, the communication efficiency of Overbot is unbearable for a botnet. An implementation which is capable of issuing 600 *get_peers* requests per second would require 5.3 hours for a 90% probability of hitting a type of nodes which collect data from bots in the Kademlia P2P network. The probable round trip time of a message of DUSTBot is less than 10 seconds. Besides, a single node which is issuing a large number of requests might be detected as a bot node in a botnet.

Lee and Kim [37] explore a new botnet with alias flux that use USSes (URL shorting service) to hide their C&C channels. The basic idea of alias flux is changing the shortened URLs associated with the obfuscated IP address of C&C servers, similar to the domain flux. A traffic monitor cannot capture the retrieved aliases and obfuscated IP address when a bot uses USSes which support HTTPS. However, to prevent abuse use, user authentication is required by some USS service providers, such as API keys or CAPTCHAs solving before shortening URLs. Besides, cooperate with companies providing USS service, the main C&C server might be tracked by defenders.

Chen *et al*. [38] propose CloudBot, which uses multiple cloud services (Baidu, Box, Dbank, Dropbox, Google Drive, and OneDrive.) as its C&C channel. The botmaster efficiently issues commands to bots through cloud-based push services and collects the data upload by CloudBots through cloud-based storage services. This solution is practical, with no limitations in terms of bandwidth, latency, and security. However, cloud service providers require user identification, including ID number or credit card number. With the information above, the botmaster is easily tracked by law enforcement. Furthermore, the botmaster might be tracked cooperating with cloud service providers if defenders capture one or more CloudBots. Since the Bitcoin network is designed to withstand these very kinds of attacks, DUSTBot cannot be shut down by regulatory processes.

Other studies towards novel C&C mechanism can be referred to in further research on botnet communication: Wang *et al*. [39] propose a stealthy email-based P2P-like botnet that exploits the excellent reputation of email servers and a considerable amount of benign email communication in the same channel to combat the detecting method based on machine learning algorithms. Desimone *et al*. [40] suggest creating covert channels in BitTorrent protocol messages. Wu *et al*. [41] propose a serverless C&C channel model using a novel strategy named Service Flux, which contains multiple subchannels. These studies present more possible threat models of botnets. However, the limitations of them are non-negligible for the botmaster in terms of invulnerability, communication efficiency, and stealthiness.

Inspired by the ideas in [24,25], we proposed DUSTBot, which enhances the upstream channel with the Bitcoin testnet to overcome the vulnerability of collecting upstream data with web servers. Since testnet Bitcoin is free to get, the scalability of DUSTBot is not limited by the price of Bitcoin. Upstream data are collected and sent back to the Botmaster by sensor bots in the proposed P2P network efficiently. The class of a single bot is decided by the Botmaster at any time via the downstream channel.

## 3. Methods

### 3.1 Botnet architecture

The proposed botnet is a bot-only P2P botnet without benign peers, which is flexible to scale. Bots in the proposed botnet are classified into two classes: sensor bot and regular bot. Each sensor bot *i* possesses two key pairs (*sk*_*i*_, *pk*_*i*_) and the derived addresses to send and receive Bitcoins. There are unspent transaction outputs (UTXO) on the Bitcoin addresses possessed by the first class of bots. Thus this class of bots is capable of sending legitimate Bitcoin transactions. The botmaster is capable of fetching the transactions sent by these bots and extract the upstream data. Thus the first class of DUSTBot is called sensor bot. The second class of DUSTBot is called regular bot since there are no Bitcoin credentials (key pairs) possessed by them. There are no differences between sensor bots and normal bots during the peer list exchange procedure and botnet propagation. Bots individually connect to the Bitcoin network and fetch the broadcast transactions in order to wait and receive the downstream data sent by the botmaster. The botmaster can easily upgrade any regular bot into a sensor bot through the downstream channel.

**Fig 1:**
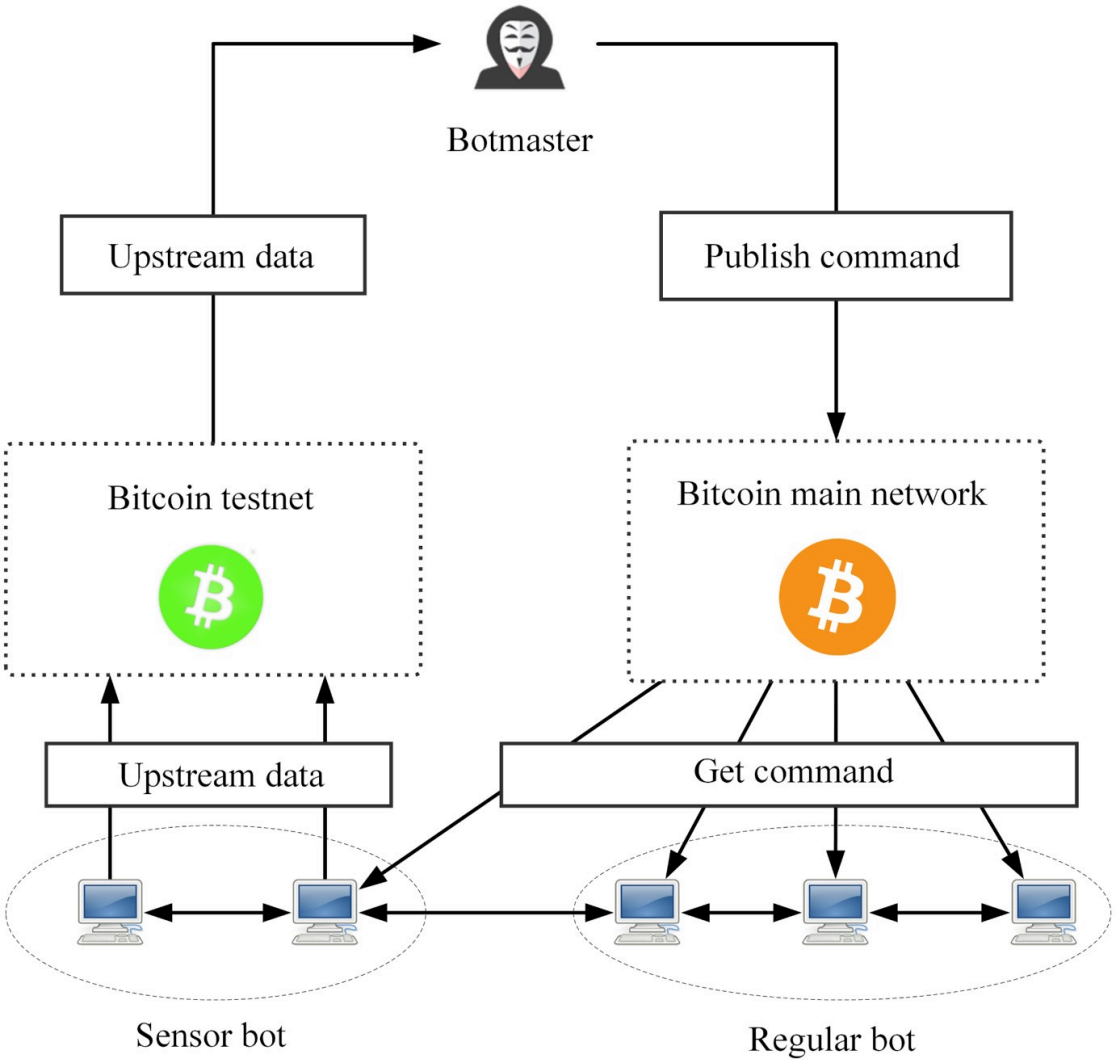
C&C architecture of the proposed P2P botnet model.

Fig 1 shows the C&C architecture of DUSTBot. The proposed P2P botnet consists of sensor bots and regular bots. All bots individually connect to the Bitcoin main network, waiting and receiving commands. The botmaster embeds commands into transactions. Bots identify these transactions by verifying the signature with the public key of the botmaster, which is hardcoded into the bot binary file. DUSTBot forwards messages from one bot to other bots until the TTL (time to live) values of the broadcast messages decrease to zero. Sensor bots collect messages from other bots, periodically issue upstream data and embed them into testnet transactions. Public keys possessed by sensor bots are known to the botmaster. Therefore, the transactions sent by sensor bots could be identified by the botmaster. Based on this, upstream data from bots could be received by the botmaster.

### 3.2 Botnet C&C mechanism

#### 3.2.1 Embedding metadata into bitcoin transactions

Utilizing the OP_RETURN output script function [31], any data could be embedded in the output script of a Bitcoin transaction [25]. This function is available after 0.9.0 version of Bitcoin Core client. Up to 83 bytes of metadata can be inserted in a single transaction. This bandwidth is sufficient for communication between botmaster and botnet as well as messages among the botnet.

#### 3.2.2 Communication between each role

**Fig 2:**
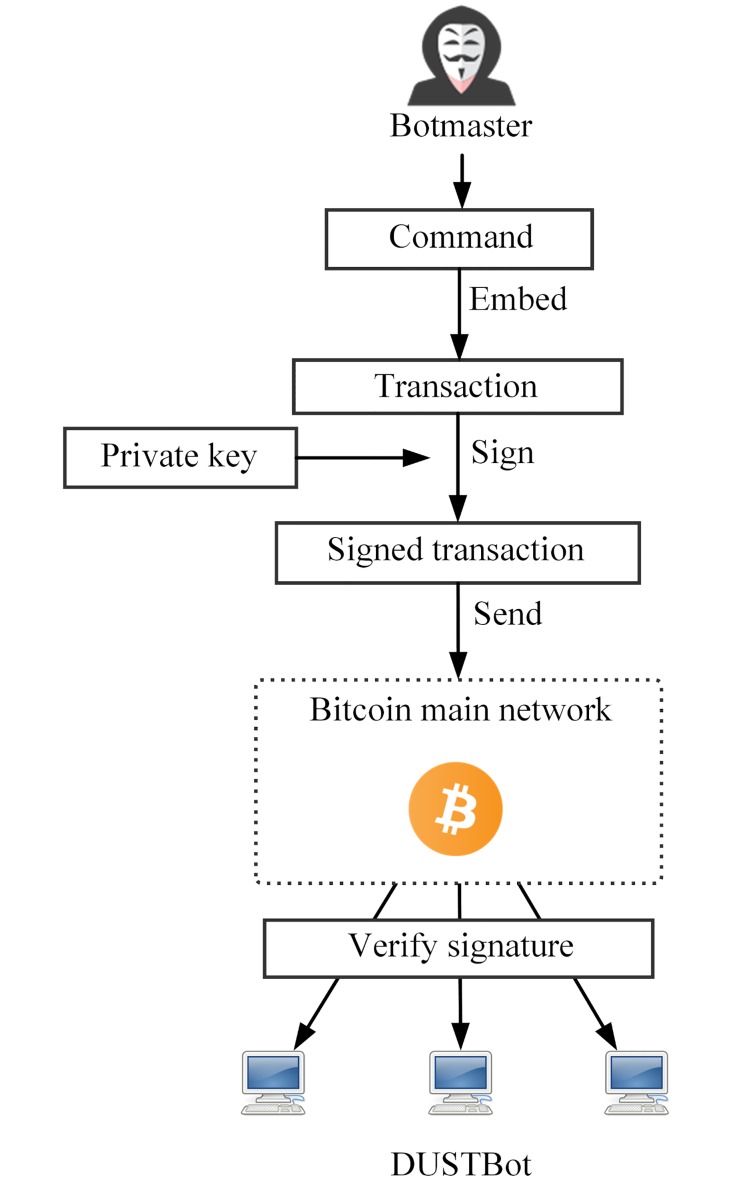
The process of downstream communication.

3.2.2.1 Downstream communication. The botmaster issues instructions via legitimate Bitcoin transactions. A DUSTBot tries fetching transactions sent by the botmaster to receive commands. The botmaster generates a key pair (*sk*, *pk*) as a set of Bitcoin credentials. The public key, *pk*, is hardcoded into the DUSTBot binary file. A transaction with the embedded command data is signed using the private key, *sk*, and then the transaction is sent. A transaction is verified as legitimate and then propagated in the Bitcoin P2P network. A DUSTBot connects to genuine Bitcoin nodes to receive the broadcast transactions, then authenticate communication from the botmaster by verifying signatures of broadcast Bitcoin transactions, then decode the instructions and execute them. The process of downstream communication is shown in Fig 2.

**Fig 3:**
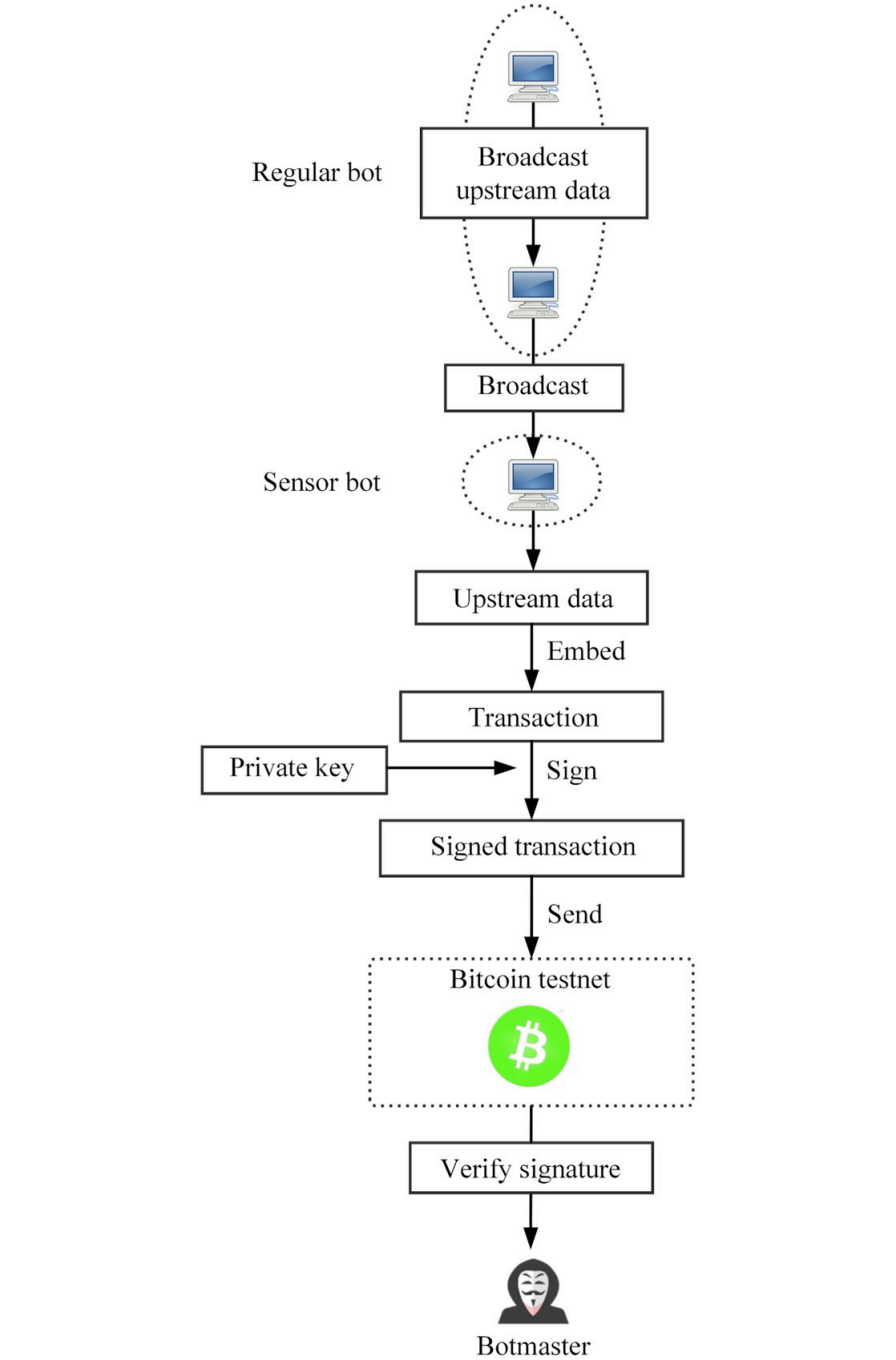
The process of upstream communication.

3.2.2.2 Upstream communication. Each sensor bot *i* possesses two key pairs (*sk*_***i*1**_, *pk*_***i*1**_, *sk*_***i*2**_, *pk*_***i*2**_) distributed by the botmaster. And two corresponding Bitcoin testnet addresses are then derived. A sensor bot embedded upstream data into a testnet transaction, then send the transaction from one of the two addresses possessed by it to another address. The botmaster connects to the Bitcoin testnet, identifies transactions sent by sensor bots, and extract the upstream data. The process of upstream communication is shown in Fig 3.

**Fig 4:**
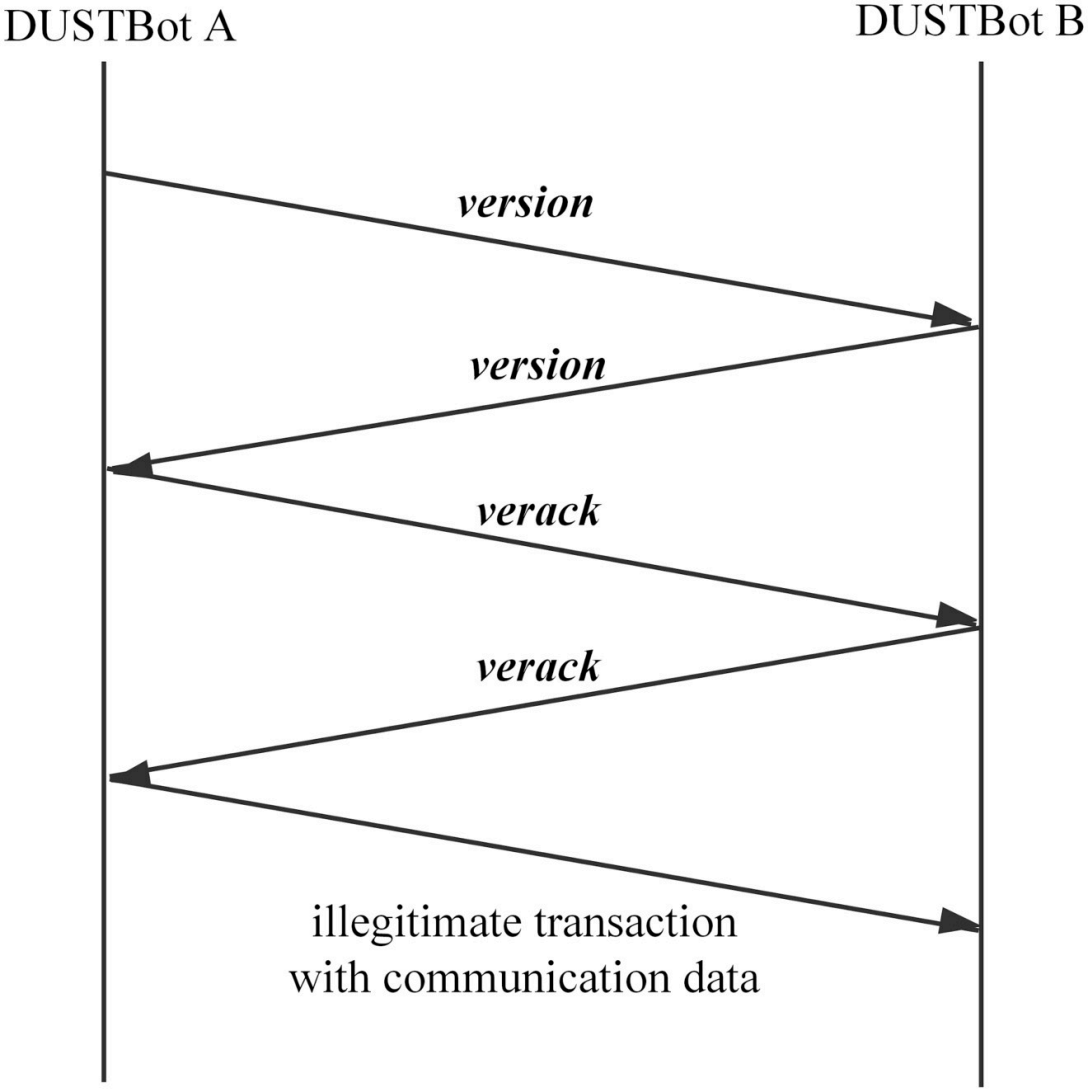
The disguised Version Handshake.

3.2.2.3 Communication among bots. Genuine Bitcoin nodes communicate with each other via Bitcoin messages [31], i.e., *version*, *verack*, *ping*, *tx*. Version Handshake is processed first in a single communication between two genuine Bitcoin nodes. Similarly, there is a disguised Version Handshake before the communication between two bots in order to check if the other bot is available and obfuscate the network traffic. Communication data is embedded into illegitimate transactions. A DUSTBot extracts communication data from the illegitimate receiving transactions. The process of a disguised Version Handshake is shown in Fig 4.

#### 3.2.3 Ethereum-block-hash-based peer list exchange algorithm

In general, botnet monitoring is crucial to execute effective takedown operations, including making an accurate botnet topology snapshot and revealing identities of bots. This is usually done by P2P botnet crawling.

3.2.3.1 State of the art. Countermeasures against monitoring aim to make it difficult for the defenders to enumerate and monitor the botnets. There are some existing anti-crawling techniques in both theoretical botnets and real-world case scenarios.

- ***Sality*** [42]: In *Sality*, bots return one randomly chosen peer from their peer list. However, by returning random peer for each query, a considerable portion of the neighbor list can be easily obtained using repeated queries.
- ***ZeroAccess*** [43,44]: In *ZeroAccess*, the *primary* peer list of bots is sorted by *most-recently* responsive peers. A bot returns the first-16 nodes (the capacity of the peer list of a bot is 256) from its peer list in a resulting reply message.
- ***P2P Zeus*** [45]: A bot of *P2P Zeus* limits the size of the returned subset to 1/5 of the capacity of its peer list. Besides, the returned subset is selected by minimal *Kademlia*-like XOR distance to the identifier of the requesting bot. However, this anti-monitoring countermeasure is effectively circumvented by the *ZeusMilker*[46] algorithm.

3.2.3.2 The proposed anti-crawling countermeasure. Karuppayah *et al*. [46] proposed a *Bit-XOR*+ algorithm which adds additional randomness at the side of the queried node. The queried node generates a random key uniformly for each IP address it receives a request from and stores it. The returned subset is biased towards a specific *XOR*-ed key generated by the random key corresponding to the IP address of the requesting bot. The algorithm proposed in [46] is effective against crawling strategies which deterministically reveal the complete peer list of a single bot and hence can efficiently provide a reliable topology snapshot of a P2P botnet. Inspired by this algorithm, we propose an Ethereum-block-hash-based peer list exchange algorithm in this subsection, which utilizes the randomness and efficiency of the latest Ethereum block hash. The detailed procedures of peer list query and reply are described in Algorithm 1 and Algorithm 2. The purpose of Algorithm 1 is to defend against routing table poisoning, and the purpose of Algorithm 2 is to mitigate the efficiency of existing crawling strategies.

**Algorithm 1** Peer list request algorithm

**Input**: *PL*_*req*_

**Output**: *L*_*ret*_

1. *N*_*need*_ ←*M* − GetSize(*PL*_*req*_)

2. **if** *N*_*need*_ = = 0 **then**

3.  **return** ∅

  // Initialization

4. *L*_*ret*_ ←∅

5. **if** | *PL*_*req*_ | = = 0 **then**

6.  *L*_*seed*_ ←GetSeedFromBitcoinBlockchain()

7.  **for** *i* = 0; *i* < *L*_*seed*_; *i* + + **do**

8.   request(*L*_*seed*_[*i*])

9. **else**

10.  **for** *i* = 0; *i* < *L*_*B*_; *i* + + **do**

11.   request(*PL*_*req*_[*i*])

12. *L*_*temp*_ ←WaitForAllResponse()

13. *H*_*Ethereum*_ ←GetEthereumBlockHash()

14. **if** *L*_*temp*_ ≠ ∅ **then**

15.  **for** *i* = 0; *i* < *L*_*temp*_; *i* + + **do**

16.   *H*_*i*_ ←SHA-256Hash(*L*_*temp*_[*i*]+*H*_*Ethereum*_)

17.   *L*_*hash*_ ←*L*_*hash*_∪{(*L*_*temp*_[*i*], *H*_*i*_)}

18.  SortByHash(*L*_*hash*_)

19.  **while** | *L*_*ret*_ | < *N*_*need*_ && | *L*_*hash*_ | > 0 **do**

20.   *L*_*ret*_ ←*L*_*ret*_∪GetIP(*L*_*hash*_[0])

21.   *L*_*hash*_ ←*L*_*hash*_ − *L*_*hash*_[0]

22.  **return** *L*_*ret*_

**Algorithm 2** Peer list response algorithm

**Input**: *PL*_*res*_, *IP*_*req*_

**Output**: *L*_*res*_

1. *L*_*res*_←∅

2. **if** IPInKeyList(*L*_*k*_, *IP*_*req*_) **then**

3.  *K*_*req*_ ←GetKey(*L*_*k*_, *IP*_*req*_)

4. **else**

5.  *H*_*Ethereum*_ ←GetEthereumBlockHash()

6.  *s*_*1*_←SHA-256Hash(*IP*_*req*_+*H*_*Ethereum*_)

7.  *s*_*2*_←SHA-256Hash(*IP*_*req*_)

8.  *K*_*req*_←XOR(*s*_*1*_, *s*_*2*_)

9. **for** *i* = 0; *i* < *S*_*return*_ && *i* <| *PL*_*res*_ |; *i* + + **do**

10.  *L*_*res*_[*i*] ←*PL*_*res*_[*i*]

11. **for** *i* = *S*_*return*_; *i* < | *PL*_*res*_ |; *i* + + **do**

12.  **for** *j* = 0; *j* < *S*_*return*_; *j* + + **do**

13.   *s*_*temp1*_←SHA-256Hash(*PL*_*res*_[*i*])

14.   *s*_*temp2*_←SHA-256Hash(*L*_*res*_[*j*])

15.   **if** XOR(*s*_*temp1*_, *K*_*req*_) < XOR(*s*_*temp2*_, *K*_*req*_) **then**

16.    *L*_*res*_[*j*] ←*PL*_*res*_[*i*]

17.    break

18. **return** *L*_*res*_

When a requesting bot is going to request peers to fulfill its peer list, the requesting bot first calculates the number of peers *N*_*need*_ to be added into its peer list by subtracting current peer list size from the capacity of a peer list *M* (Line 1). If *N*_*need*_ is 0, an empty set is returned (Line 2 and Line 3). Then, if there are no peers in its peer list, it would try sniffing the Bitcoin blockchain to get seed peers and retrieve peers to fulfill its peer list (Line 5 to Line 8). Seed peers are released and periodically updated to the Bitcoin blockchain via transactions corresponding to a specific Bitcoin address.

Otherwise, it retrieves peer from existing peers in its peer list (Line 10 to Line 11). After collecting all the responses into a temporary IP list *L*_*temp*_, candidates to be added into the peer list are selected by a filtering procedure. It first tries sniffing the Ethereum blockchain to get the latest Ethereum block hash *H*_*Ethereum*_ (Line 13). We use Ethereum instead of Bitcoin because Ethereum is much faster to generate a new block. The average time of generating a new Ethereum block is about 20 seconds, while the average time of generating a Bitcoin block is about 10 minutes [28]. Then the SHA-256 hash value *H*_*i*_ of every single IP address *L*_*temp*_[*i*] in *L*_*temp*_ combined with *H*_*Ethereum*_ is generated (Line 16). The IP address *L*_*temp*_[*i*] and the corresponding hash value *H*_*i*_ are then collected into a result list *L*_*hash*_ (Line 17). After that, *L*_*hash*_ is sorted in ascending or descending order, and the first *N*_*need*_ results are added to the return list *L*_*ret*_ (Line 18 to Line 21). This may resort to a biased peer list since peers with either the highest or lowest hash value would be preferably returned. The bias will be evaluated through an experiment in Section 4.4.

When the queried node receives a request from another node, the queried node will return a biased subset of size *S*_*return*_. This is an effective countermeasure to restrict the efficiency of P2P botnet crawling. Inspired by [46], we employ a similar way in Algorithm 2 at the side of the queried node, which adds additional randomness for the queried node to return peers. The queried node generates a SHA-256 hash value of the requesting IP address *IP*_*req*_ combined with the latest Ethereum block hash *H*_*Ethereum*_ as the unique key *s*_*1*_ (Line 5 to Line 6). Then the key is *XOR*-ed with the SHA-256 hash value *s*_*2*_ of the requesting IP address *IP*_*req*_ and the unique resulting key *K*_*req*_ is stored (Line 7 to Line 8). After that, the returned peers are selected by minimal XOR distance to *K*_*req*_. It first constructs a list *L*_*res*_ containing up to the first *S*_*return*_ peers in its peer list *PL*_*res*_ (Line 9 to Line 10). Then it iterates over *PL*_*res*_ (Line 11). The XOR distance of the SHA-256 hash value of each *PL*_*res*_[*i*] to *K*_*req*_ is compared to the XOR distance of the SHA-256 hash value of each *L*_*res*_[*j*] to *K*_*req*_ (Line 15). Once a *PL*_*res*_[*i*] with smaller XOR distance to *K*_*req*_ than *L*_*res*_[*j*] is found, *L*_*res*_[*j*] is replaced with *PL*_*res*_[*i*] (Line 16).

The proposed peer list exchange algorithm is effective against routing table poisoning attack and P2P botnet crawling. First, utilizing the randomness the latest Ethereum block hash in Algorithm 1, each element in *L*_*temp*_ has an equal likelihood to be added into the peer list of the requesting node. This countermeasure provides more challenges for defenders to inject nodes into peer lists of bots unless the defenders are capable of realizing a 51% attack [47] towards a blockchain network. However, theoretically, the cost of a 51% attack on Ethereum network is unbearable for individuals [48]. Second, the returned subset of the queried node is biased to the specific *XOR*-ed key corresponding to the IP address of the requesting node. Hence, a fixed subset is returned to the same requesting IP address. The efficiency of botnet crawling against the DUSTBot is restricted. Moreover, defenders are not able to work out a further analysis through the results of peer list exchange.

#### 3.2.4 Seed peers on the bitcoin blockchain

Seed peers are released and periodically updated on the Bitcoin blockchain via transactions corresponding to a specific address. A bot without peers in its peer list tries sniffing the Bitcoin blockchain and retrieves entries into the DUSTBot P2P network. After that, it requests more peers from bootstrap nodes to fill the peer list until it reaches its capacity *M*. It is hard for defenders to block Bitcoin transactions even if they work out the Bitcoin address possessed by the botmaster since it is hard to reach a consensus over numerous miners.

## 4. Experiments and results

To proof the concepts we proposed in Section 3, several experiments are carried out in this section. In Section 4.1 and Section 4.2, we first construct a botnet through a P2P simulator, then a real P2P botnet which consists of prototypes is deployed on cloud platforms based on the results of the simulation. As metrics, we measure the feasibility and performance by the *round-trip time* (RTT) over the DUSTBot network between the botmaster and DUSTBot. In Section 4.3, we measure the robustness of DUSTBot by calculating the *connected ratio* after removing a different fraction of sensor bots.

### 4.1 Botnet construction

In this subsection, we first construct the botnet according to the properties defined in Table 1 with a P2P simulator. We measure the success of botnet construction by network properties we observed, which is presented in Table 2.

**Table 1:** The definition of network properties.

Property	Description
*N*_*h*_	Total number of hosts
*J(t)*	Number of infectious hosts at time *t*
*β*	The infection rate of botnet propagation in each time interval
*N*	The population of the current botnet
*M*	The capacity of the peer list of a bot
*μ*_*out-degree*_	The average number of the out-degree of a bot
*D*	The diameter of the simulated botnet (the maximum path length from a regular bot to a sensor bot)
*μ*_*pathlen*_	The average path length from a regular bot to a sensor bot
*p*_*init*_	Initial proportion of sensor bots
*p*_*sensor*_	The current proportion of sensor bots
*N*_*sensor*_	The current number of sensor bots
*TTL*_*up*_	The hop limit of a broadcast message
*S*_*peer*_	The number of peers that a peer list currently contains
*μ*_*peer*_	The average number of peers that a peer list currently contains
*S*_*sensor*_	The number of sensor bots that a peer list currently contains
*μ*_*sensor*_	The average number of sensor bots that a peer list currently contains

**Table 2:** Properties of the constructed P2P botnet.

Property	Value
*N*	20000
*N*_*sensor*_	4947
*μ*_*out-degree*_	9.9
*μ*_*peer*_	19.9
*μ*_*sensor*_	6.1
*D*	5
*μ*_*pathlen*_	0.9

#### 4.1.1 Network properties definition

We define the properties of DUSTBot in Table 1.

#### 4.1.2 Construction procedure

We construct a botnet with PeerSim [49], an open source P2P simulator. There is no bootstrap procedure for DUSTBot. This avoids the bootstrap vulnerability. Wang *et al*. [50] and Liu *et al*. [51] employ the new infection and reinfection mechanism to propagate botnet. We use a similar mechanism to construct peer lists. Assume that the capacity of the peer list of a DUSTBot is *M*. If a vulnerable host *B* is infected by a bot *A*, *A* passes its peer list to the newly infected host *B*, and *B* will also add *A* into its peer list. When bot *B* is reinfected by bot *A*, *R* (*R*<*M*) randomly selected peers in the peer list of *B* are replaced by *R* peers in the peer list passed by *A*. Also, *A* and *B* will add each other into their peer lists. The reinfection procedure can effectively interconnect different infection paths together, making a botnet evenly connected.

Scanning and vulnerability exploit is the dominant infection mechanism. Thus the simulation of our botnet construction is similar to worm propagation. Epidemic models are applied to model computer virus, and worm propagation since the propagation of worms is similar to the biological infectious diseases.

Classical simple epidemic model [52] is employed in our botnet construction. In this model, each host stays in one of two states: susceptible or infectious. Hosts that are vulnerable to be infected are called *susceptible* hosts; hosts that have been infected and can infect other hosts are called *infectious* hosts. We assume that a host will stay in the infectious state forever once it is infected by an infectious host. The classical simple epidemic model for a finite, vulnerable population is Eq (1).

$$\frac{dJ(t)}{dt}=\beta J(t)[N_{h}-J(t)]$$ (1)

where *J(t)* is the number of infectious hosts at time *t*; *N*_*h*_ is the sum of infectious and susceptible hosts, and *β* is the infection rate.

#### 4.1.3 Initialization

As indicated in [50], botnets kept their populations to an average of 20000. In Eq (1), when *t* = 0, *J*(0) hosts are infectious, and the other *N*_*h*_ − *J*(0) hosts are all susceptible. Suppose the size of *N*_*h*_ is 20000, *N* stops growing after all the susceptible are infected. *β* is defined by a parameter *k* where *k* = *βN*_*h*_. In this paper, *J*(0) is configured to 21, and *k* is configured 1.8 as what used in [50] and [52]. Besides, we assume that all the initial infectious hosts are sensor bots. We set *M* to 20 for comparison to [50]. Then the population of the constructed botnet grows to 20000 continuously during the simulated botnet propagation. Regular bots are also updated to sensor bots continuously to keep the value of *p*_*sensor*_ to about 0.25. After initialization, *μ*_*sensor*_ is defined as Eq (2).

$$\mu_{sensor}=\frac{1}{N}\sum_{i=1}^{N}S_{sensor_{i}}$$ (2)

Where $S_{sensor_{i}}$ is the number of sensor bots contained in the peer list of bot *i*. The summary of the simulated P2P network is provided in Table 2.

**Fig 5:**
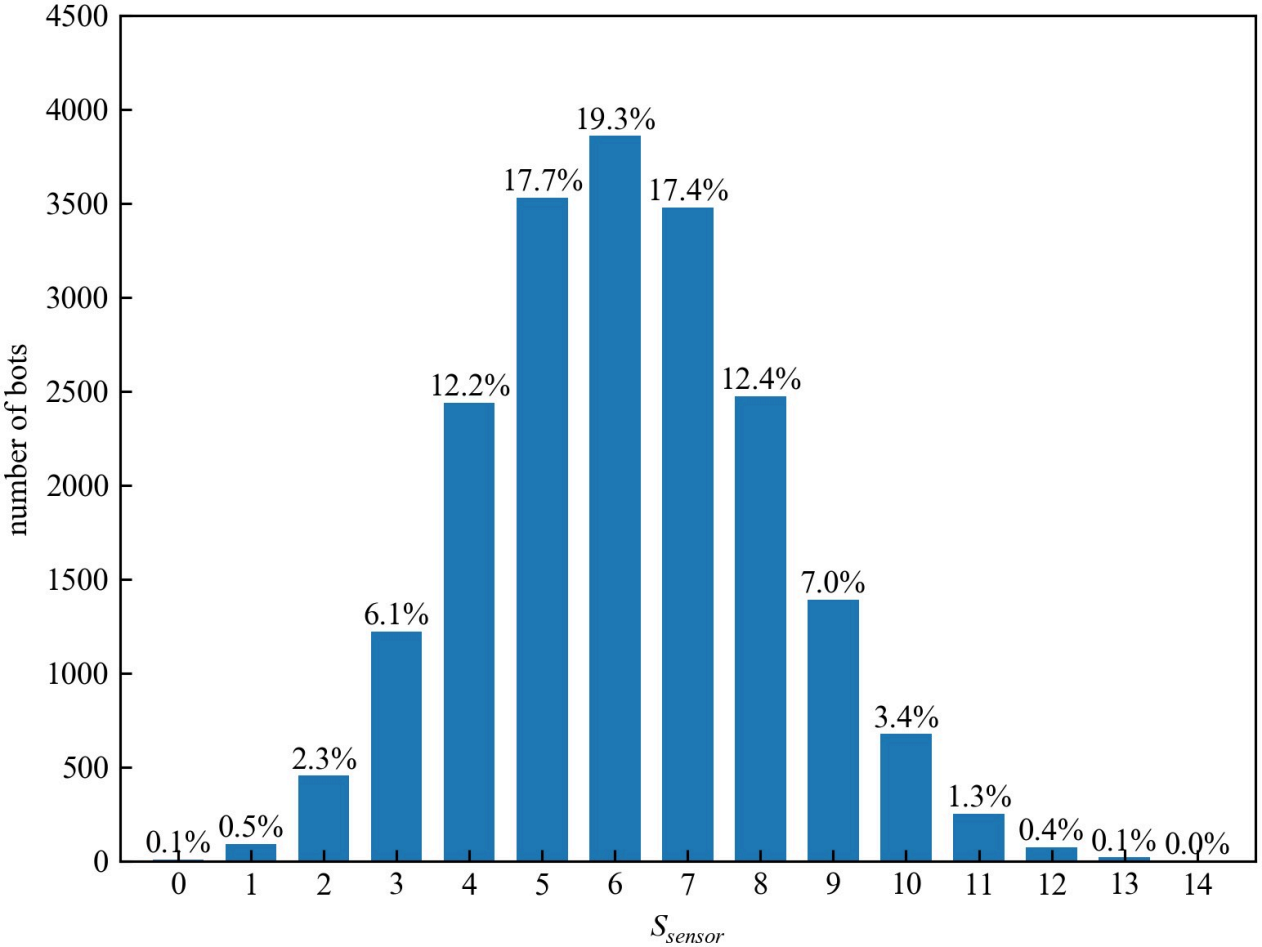
The distribution of the current number of sensor bots in peer lists.

The value of *μ*_*sensor*_ we observed in the simulated network is 6.1. During the botnet propagation, overall, about 180000 reinfections occurred. Fig 5 shows the distribution of *S*_*sensor*_ in the simulated botnet. The value of *S*_*sensor*_ distributes from 4 to 8 over 80% of bots. The distribution of *S*_*sensor*_ roughly follows a normal distribution. Hence, the connectivity of the simulated botnet is well-balanced. To study the dispersion degree of *S*_*sensor*_, we calculate the standard deviation of *S*_*sensor*_. Assume the standard deviation of *S*_*sensor*_ is denoted by *σ*, *σ* is defined as Eq (3).

$$\sigma =\sqrt{\frac{1}{N-1}\overset{N}{\underset{i=1}{\sum }}{\left(S_{sensor_{i}}-\mu_{sensor}\right)}^{2}}$$ (3)

**Fig 6:**
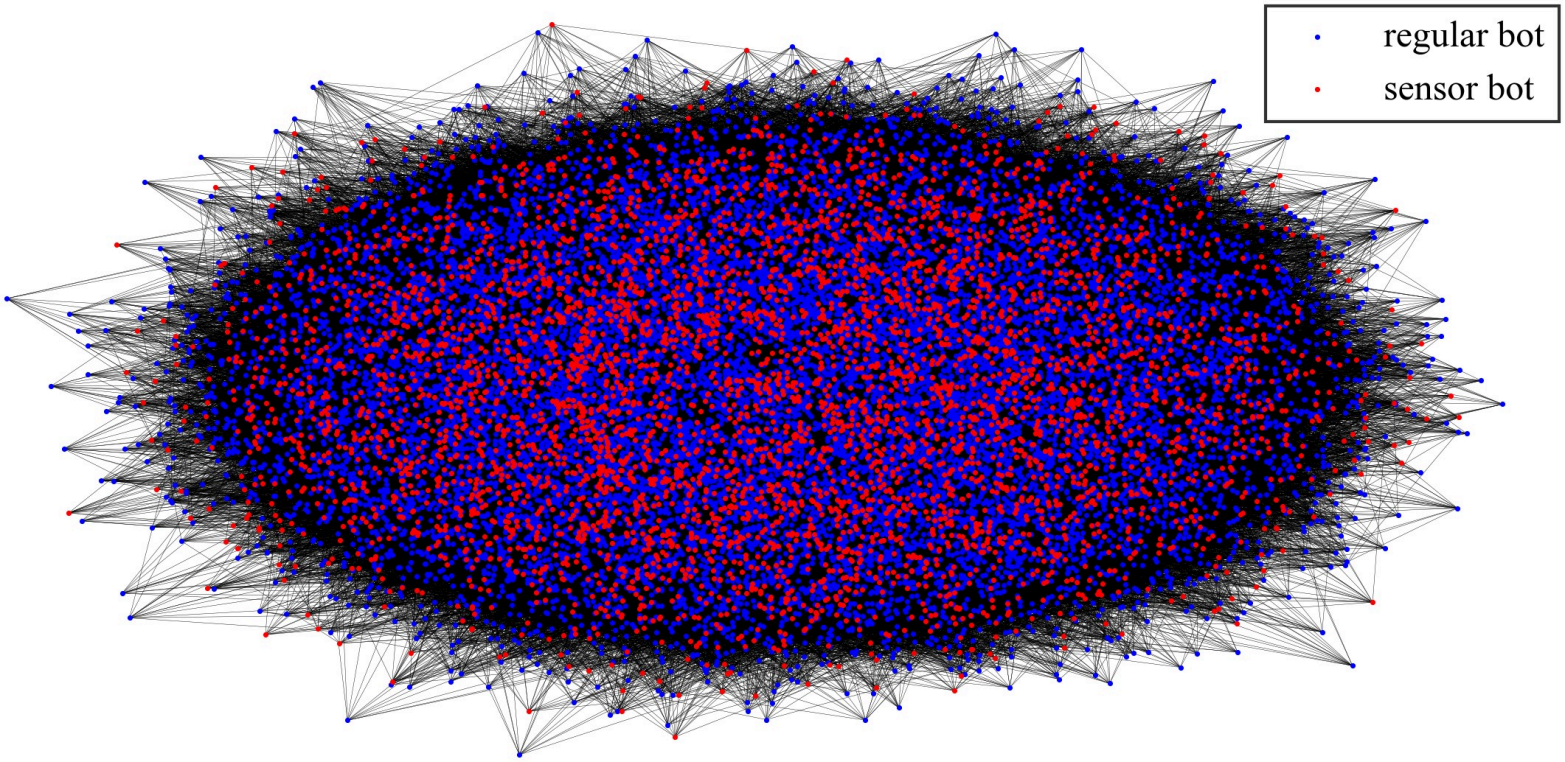
A network overview of the simulated botnet.

The value of *σ* we observed in the simulated network is 2.012, which indicates that there is a dispersion among *S*_*sensor*_. This may affect the maximum and the average path length *D* and *μ*_*pathlen*_ from a regular bot to a sensor bot. The value of *D* is 4, and the value of *μ*_*pathlen*_ is 0.9. *D* and *μ*_*pathlen*_ represent the connectivity between the regular bots and the sensor bots. This may affect the *reachable ratio* of the constructed botnet. The *reachable ratio* represents the probability that a communication message reaches an available sensor bot after the broadcast through *TTL*_*up*_ hops. The *reachable ratio* would be studied in Section 4.3. Fig 6 shows the network overview of the simulated botnet, red dots represent sensor bots, and blue dots represent regular bots. Fig 6 shows that all the bots are uniformly distributed.

### 4.2 DUSTBot on cloud platforms

#### 4.2.1 Experimental setup

**Fig 7:**
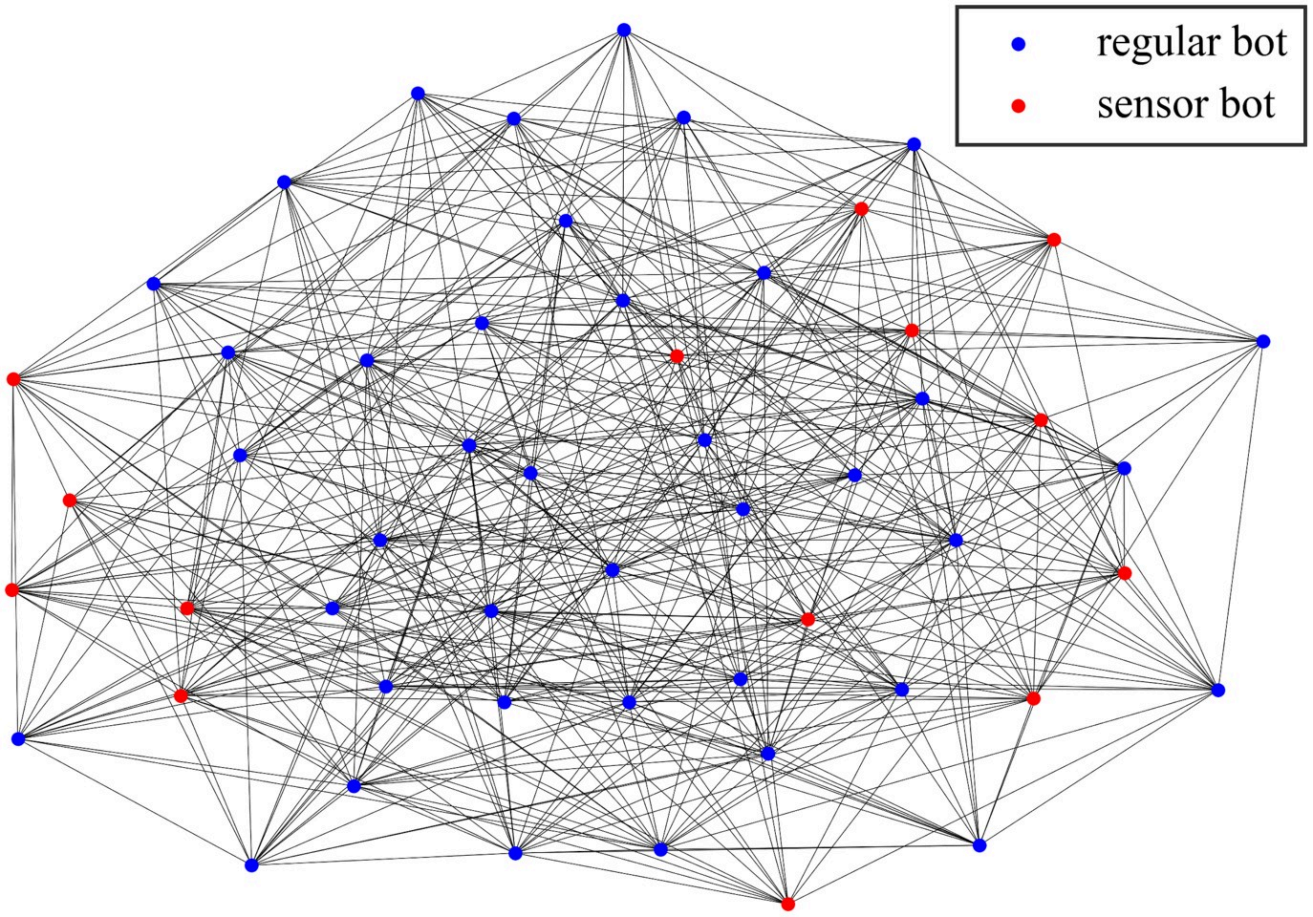
A network overview of the pre-constructed P2P botnet deployed on cloud platforms.

To implement the solution we proposed, we use the BitcoinJ library [53], an open source Java API (Application Programming Interface) of the Bitcoin protocol. The executable of DUSTBot prototype is 12.9MB in size. Inspired by [24,25], we deploy a similar pre-constructed P2P network on Microsoft Azure cloud platform and Amazon Web Service to test the feasibility and performance of DUSTBot. Due to the restriction of cloud platforms, only 54 nodes are deployed, including 40 nodes on Amazon Web Service and 14 nodes on Microsoft Azure. For every single node, the system is Ubuntu Server 18.04 LTS, the RAM (Random Access Memory) is 1GB, and the number of virtual CPU (Central Processing Unit) is 1. Network bandwidth of these nodes is not provided by the cloud platforms. Bots connect to the Bitcoin main network, try identifying transactions from the botmaster in the broadcast transactions, and then extract the metadata embedded in the identified transactions. Refer to the parameters of the simulation, 14 of all 54 nodes are deployed as sensor bots. Fig 7 shows the network overview of the pre-constructed P2P botnet deployed on cloud platforms, red dots represent sensor bots, and blue dots represent regular bots.

#### 4.2.2 Metrics

As metrics, we measure the feasibility and performance by the *round-trip time* (RTT) over DUSTBot network between the botmaster and DUSTBot. The RTT includes the delay of 1) a DUSTBot captures transaction from the botmaster; 2) the broadcast of upstream data in DUSTBot P2P network; 3) the botmaster captures transaction from a sensor bot. In order to test the feasibility and performance of the proposed C&C channel, modules which may lead to peer discard are removed in the executable we deployed in this experiment. Besides, we use the Bitcoin testnet as the downstream channel under the consideration of the economic cost in this experiment (there is not much difference in the efficiency of transaction broadcast). To avoid too many redundant responses, the TTL value is set to 1 in this experiment.

#### 4.2.3 Results

**Fig 8:**
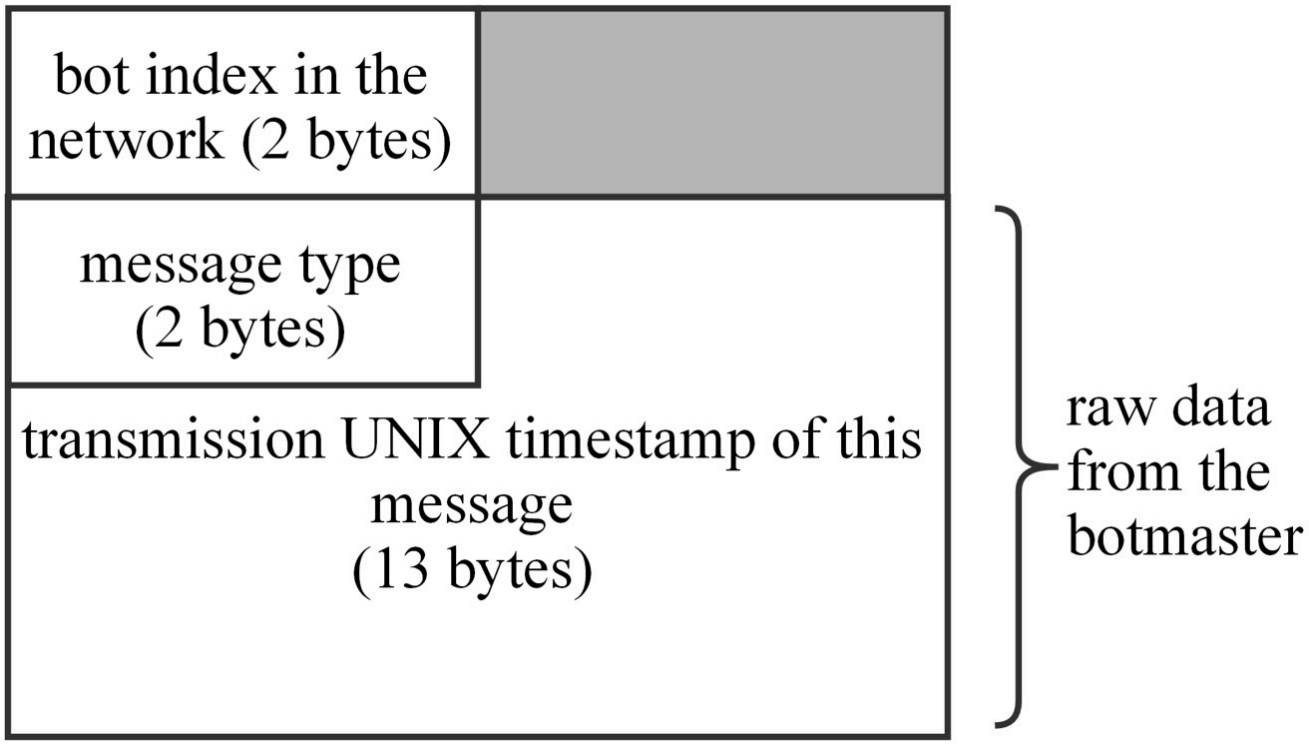
The data structure of the upstream message.

According to Fig 7, the average number of sensor bots in a single peer list of regular bots is 2.5. 100 responses are expected to be received in a single command issue, and the redundant responses from the same regular bot are filtered. We send data through Bitcoin transactions every 40 minutes for over 48 hours. 73 transactions are sent totally. Fig 8 shows the data structure of communication messages.

When bots capture the transaction, they send the data and their identities back through the upstream channel except for sensor bots. 7293 of 7300 responses are received in this experiment. The response rate is 99.904%. Besides, all 2920 valid responses are received in this experiment. The RTTs of all bots varies from 2.169 to 23.367s with an average of 6.8298 and standard deviation of 2.4718. Due to the connectivity of the Bitcoin P2P network, about 50% of the commands, the bots respond within 6s, and 90% of the commands within 10s. In [24,25], 50% of the bots of ZombieCoin respond within 5s, and 90% of the commands within 10s. However, the upstream channel of ZombieCoin is not the Bitcoin P2P network, but a traditional web server owned by the botmaster. Hence, the RTT of DUSTBot is expected to be larger than ZombieCoin. In [30], the average RTT of UnblockableChains is about 4s. The C&C infrastructure of BOTCHAIN [26] is similar to DUSTBot except for the upstream channel. Thus, the RTT of BOTCHAIN is expected to be close to DUSTBot. Therefore, the network latency of the proposed C&C channel is similar to existing solutions which build their C&C channels on public blockchains. And the latency of the proposed C&C channel is acceptable for a botnet. The detailed experimental data in this subsection is given in the Table A in S1 Appendix, including the Bitcoin testnet addresses we used, the first and final transaction id and index in the Bitcoin testnet blockchain.

### 4.3 Botnet robustness evaluation

In this subsection, we will evaluate the robustness of the proposed botnet model. Many factors affect the robustness of a botnet, i.e., removal of sensor bots, DDoS attack, Sybil attack, routing table poisoning, and peer off-line. Those factors have the same impact on the connectivity of a botnet.

#### 4.3.1 Metrics

4.3.1.1 Robustness Metric Function. Botnet connectivity is a considerable measure to express the botnet robustness. We use the following metric function presented in [50]. *C*(*p*_*r*_) denotes the *connected ratio* of bots to available sensor bots after removing *p*_*r*_ fraction of sensor bots in the constructed botnet, which represents the connectivity of the proposed botnet. *C*(*p*_*r*_) is defined as Eq (4).

$$C(p_{r})=\frac{N_{connected}}{N_{remaining}}$$ (4)

where *N*_*connected*_ denotes the number of bots which is connected to at least an available sensor bot, *N*_*remaining*_ denotes the number of remaining bots. *C*(*p*_*r*_) represents the connectivity of the current botnet.

4.3.1.2 Robustness Mathematical Analysis. We also provide an analytical study of the botnet robustness. The formula of *C*(*p*_*r*_) when randomly removing *p*_*r*_ fraction of sensor bots is provided. The *connected ratio* of the proposed botnet is the probability that an upstream message from a regular bot can reach an available sensor bot after broadcast. To provide a formula of *C*(*p*_*r*_), we need to calculate two parameters first: *μ*_*peer*_ and *p*_*sensor*_. *μ*_*peer*_ denotes the average number of peers that a peer list currently contains. *p*_*sensor*_ denotes the current proportion of sensor bots in the constructed botnet. *μ*_*peer*_ is defined as Eq (5).

$$\mu_{peer}=\frac{1}{N}\sum_{i=1}^{N}S_{peer_{i}}$$ (5)

where *N* is the population of the current botnet, $S_{peer_{i}}$ is the number of peers that the peer list of bot *i* currently contains. *p*_*sensor*_ decreases when sensor bots are removed. *p*_*sensor*_ is defined as Eq (6).

$$p_{sensor}=\frac{p_{init}\left(1-p_{r}\right)}{1-p_{init}\cdot p_{r}}$$ (6)

Now we discuss the probability of whether an upstream message from a regular bot will reach an available sensor bot or not. The calculation value of *C*(*p*_*r*_) could be calculated by subtracting the probability that a message cannot reach an available sensor bot from 1. First, the probability that a DUSTBot is not an available sensor bot is (1 − *p*_se*nsor*_), and the probability that all the peers contained in the peer list of the DUSTBot are not available sensor bots is ${\left(1-p_{\text{se}nsor}\right)}^{\mu_{peer}}$. Therefore, the calculation value of *C*(*p*_*r*_) is $1-(1-p_{\text{s}ensor})\cdot {\left(1-p_{sensor}\right)}^{\mu_{peer}}$ when *TTL*_*up*_ is 1. According to this, the *C*(*p*_*r*_) can be defined as Eq (7):

$$C(p_{r})=1-{\left(1-p_{sensor}\right)}^{\sum_{j=0}^{TTL_{up}}\mu_{peer}{}^{j}}$$ (7)

#### 4.3.2 Results

Eq (7) indicates that *TTL*_*up*_ has a decisive impact on *C*(*p*_*r*_) because of the exponential explosion. When *M* is 20, *p*_*init*_ is 0.25, *C*(*p*_*r*_) is close to 1 as the value of *TTL*_*up*_ increases. Thus, we need to select the optimal value of *TTL*_*up*_ through a simulation to avoid creating a broadcast storm.

The peak in the path length from a regular bot to a sensor bot would be instrumental in guiding the choice of *TTL*_*up*_. Hence, the optimal choice of *TTL*_*up*_ would be the path length value that most frequently occurs in the constructed botnet.

Next, we remove sensor bots in the constructed botnet with different fraction and observe the changes in the peak values of the path length. We assume that all sensor bots are available before they are removed. The random removal experiments are deployed to the simulated botnet we constructed in Section 4.1. The range of *p*_*r*_ is 0 to 0.95, with an interval of 0.05. Table 3 shows the peak value of the path length and its fraction among all the bots under different *p*_*r*_.

**Table 3:** Peak value and max value of the path length from a regular bot to a sensor bot under different pr.

*p*_*r*_	Peak Value	Fraction	*D*
0.0	1	61.8%	5
0.05	1	61.0%	5
0.10	1	60.3%	5
0.15	1	59.6%	6
0.20	1	58.5%	6
0.25	1	57.6%	6
0.30	1	55.4%	6
0.35	1	54.5%	6
0.40	1	53.7%	6
0.45	1	51.6%	6
0.50	1	49.8%	6
0.55	1	47.5%	7
0.60	1	43.0%	7
0.65	1	42.8%	7
0.70	1	39.1%	8
0.75	1	34.3%	9
0.80	1	28.1%	9
0.85	2	27.4%	10
0.90	2	25.3%	12
0.95	2	23.5%	14

According to Table 3, as the fraction of sensor bots in the constructed botnet decreases, the peak value of the path length from a regular bot to a sensor bot increases from 1 to 3. The fraction of each peak value decreases as *p*_*r*_ increases. Besides, the diameter of the constructed botnet also increases from 5 to 18.

Next, we remove sensor bots with different fraction and observe the changes of *C*(*p*_*r*_) calculated by the Eq (4) to evaluate the impact of the hop limit of an upstream message to *C*(*p*_*r*_). The range of *TTL*_*up*_ is 1 to 2, with an interval of 1. The range of *p*_*r*_ is 0 to 1, with an interval of 0.05.

**Fig 9:**
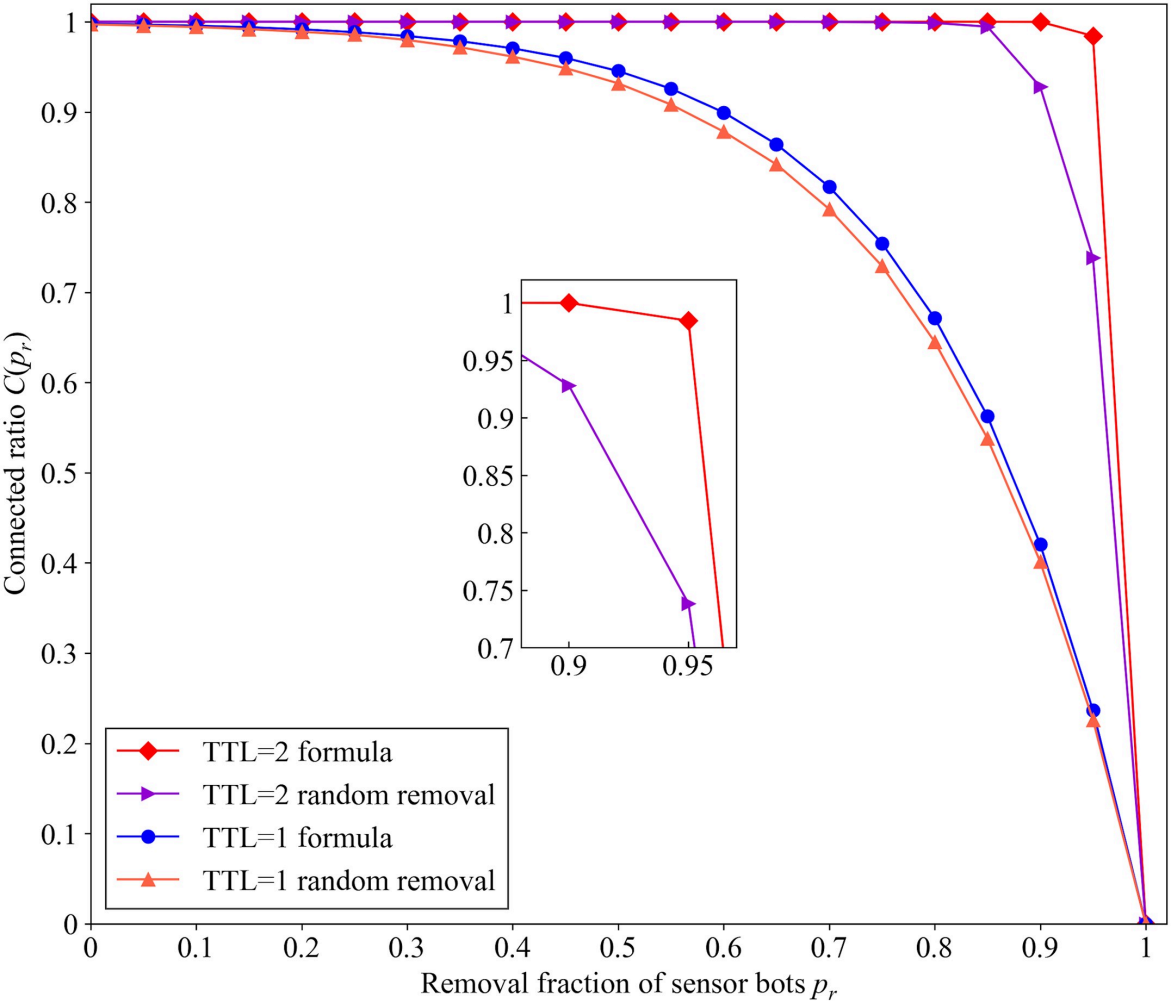
Comparison of the Eq (7) and simulation results with different *TTL*_*up*_.

Fig 9 shows the results calculated by Eq (7) in the constructed botnet, compared with the simulation result *C*(*p*_*r*_) of the random removal experiment under different values of *TTL*_*up*_. The subfigure of Fig 9 in the center is a partial enlargement of Fig 9. In Fig 9, the simulation results are slightly lower than the calculated values, since we use *μ*_*peer*_ in Eq (7), and there is a dispersion degree among all the *S*_*sensor*_, as we have discussed in Section 4.1. There is an experimental error between the calculation result and the simulation result. Although we cannot accurately evaluate the robustness of the proposed botnet via Eq (7), it provides an approximate estimate without monitoring the proposed botnet.

Fig 9 shows that the *connected ratio* is close to 1 when *TTL*_*up*_ is 2, and the range of *p*_*r*_ is 0 to 0.85. Besides, the simulation result of *C*(*p*_*r*_) is over 0.9 after removing 90% of sensor bots when *TTL*_*up*_ is 2. Hence, over 90% of the bots are still capable of communicating with the botmaster after 90% of the sensor bots are removed when *TTL*_*up*_ is 2. When the hop limit of the upstream messages is 2, the proposed botnet shows outstanding robustness after most of the sensor bots is removed. So we select 2 as the optimal value of *TTL*_*up*_. The detailed experimental configuration for this subsection is provided in S1 Appendix.

### 4.4 Evaluation of the proposed peer list exchange algorithm

In this subsection, we will evaluate the performance of the proposed peer list exchange algorithm against P2P botnet crawling and routing table poisoning attack. The detailed experimental configuration for this subsection is provided in S1 Appendix.

#### 4.4.1 Churn effects

A botnet overlay experiences high *churn* rate of nodes joining and leaving the network at high frequency [44]. Crawlers that crawl bots with a low frequency or a long duration may introduce a significant network *bias*. Moreover, bots considered to be online may have already gone offline. In addition, newly infected hosts might be missed by the crawler [44]. Accuracy of botnet snapshots produced by crawlers suffers from churn effects. To avoid the churn effect in the evaluation of proposed peer list exchange algorithm, we assume that no new bots would join or leave the constructed botnet during crawling.

#### 4.4.2 Effectiveness against botnet crawling

A P2P botnet is effectively taken down if a complete snapshot is produced by botnet crawling. To reduce the efficiency of P2P botnet crawling, we propose a specific peer list exchange mechanism in Section 2. We will evaluate the performance of the proposed peer list exchange algorithm against botnet crawling from two aspects.

4.4.2.1 Full crawl. The target of a full crawl is to discover all contactable nodes in the network.

4.4.2.1.1 State of the art. The following crawling techniques have been utilized in crawling real unstructured P2P botnets or file-sharing systems. Most of the existing crawling techniques usually implemented either a DFS (Depth-First Search) or BFS (Breadth-First Search)-based queue implementation as a node selection strategy [54].

- ***BFS-based crawler***: Holz *et al*. [19] enumerate the *Storm* botnet with a BFS-based crawler that iteratively queries each peer starting from a seed list. Rossow *et al*. [55] implement a BFS-based crawler which aims at *P2P Zeus* botnet. It starts the crawling from a seed node and appends undiscovered nodes at the end of a queue.
- ***DFS-based crawler***: Dittrich *et al*. [56] enumerate the Nugache botnet with a DFS-based crawler which conducts pre-crawls and utilizes that information as an input for their priority-queue.
- ***Less Invasive Crawling Algorithm (LICA)*** [54]: *LICA* is a generic crawling algorithm that aims to effectively enumerate the whole botnet population with queries as few as possible.

4.4.2.1.2 Metrics. To measure the performance of the anti-crawling countermeasures, we use *discovery ratio* as what used in [46] and [54]. It is defined by the fraction of peers discovered during the P2P botnet crawling. The *discovery ratio* is a metric for both the efficiency of the crawling algorithm as well as the effectiveness of the botnet’s countermeasures. Different crawling and anti-monitoring strategies could be compared via the *discovery ratio*.

4.4.2.1.3 Experimental Setup. As a bot in *P2P Zeus* usually restricts the size of the returned peer list to 10 when the size of its peer list is 50 [46]. The size of returned subset *S*_*return*_ is restricted to *M*/5 in our experiments.

We compare the effectiveness of the proposed anti-crawling countermeasure to the existing countermeasures we stated in Section 3.2.3 by crawling the constructed botnet with *BFS*, *DFS*, and *LICA*. A bot returns peers with different anti-crawling strategies when it receives a peer list request. Overall, 12 experiments are carried out. Every single experiment repeats for 50 times by choosing 50 different seed peers, and the results are averaged. Only one seed peer is chosen during each trial. We assume that all the requests are sent by the same node, and all the peer list does not change while crawling.

For *LICA*, we choose the same parameters as used in [54]. Parameter *r* is the maximum number of requests allowed to be sent to the same seed peer. Parameter *w* is the number of subsequent requests for which gain is calculated. After every *w* requests, the accumulated gain within the past *w* requests is checked. If observed gain per request drops below the threshold *t*, *LICA* may repeat another iteration of the crawl. The value of *r* is configured to 2, the value of *w* is configured to 300, and the value of *t* is configured to 0.1 in this experiment refer to [54].

Besides, to deploy the anti-crawling strategy of *ZeroAccess* for comparison, we construct a specific botnet of *ZeroAccess*, since a *ZeroAccess* bot sorts its peer list by *most-recently* responsive peers after inserting a new peer into its peer list [44].

**Fig 10:**
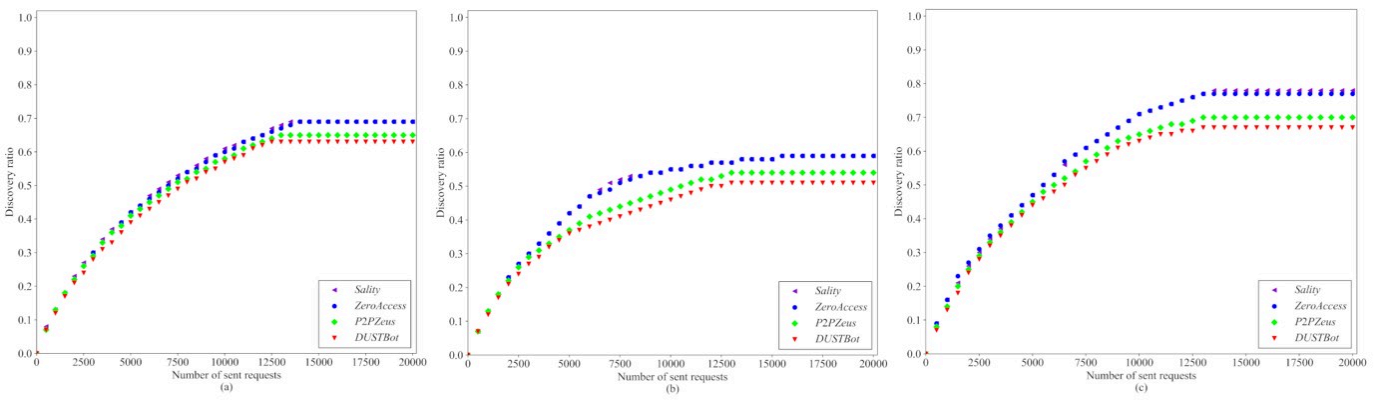
Performance of *BFS* (a), *DFS* (b) and *LICA* (c) on different anti-crawling strategies (*S*_*return*_ = 4, *M* = 20).

4.4.2.1.4 Results. Fig 10 shows the performance of existing crawling methods on DUSTBot and other existing botnets. It is expected that *Sality* and *ZeroAccess* perform worse than *P2P Zeus* and *DUSTBot* since the returned peers are biased towards the unchanged requesting node. DUSTBot is expected to perform similar to *P2P Zeus*. *LICA* performs better than *BFS* and *DFS* since it prioritizes popular nodes during the crawling [54].

As indicated in Fig 10(a), 10(b) and 10(c), *P2P Zeus* and *DUSTBot* are more effective than *Sality* and *ZeroAccess* against existing crawling strategies. DUSTBot performs slightly better than *P2P Zeus*. For the proposed anti-crawling strategy, about 67% coverage of the peers in the constructed botnet is obtained by *LICA* after 20000 requests, while 78% of the peers is obtained for *Sality*. Fig 10(a) shows that only 51% coverage of the peers is discovered by *DFS* when the anti-crawling strategy of DUSTBot is deployed.

The *discovery ratio* of *LICA* grows much slower after a large number of requests, since the researchers who proposed *LICA* assume that the complete peer list can always be obtained in each response. However, only *M*/5 of the peers in a peer list would be returned in a single peer list response. Hence, *LICA* would not be effective for the botnet proposed in this paper.

4.4.2.2 Crawling the complete peer list of a single bot. Some existing crawling approaches aim at retrieving a complete peer list of every single bot and hence can efficiently provide a reliable topology snapshot of P2P botnets.

4.4.2.2.1 State of the art. Existing crawling methods are stated as follows:

- ***ZeusMilker*** [46]: *ZeusMilker* is a novel crawling algorithm that aims to circumvent the restriction of a peer list response algorithm which biases the returned peer list to a specific key. It strategically spoofs keys to milk all peers from the peer list of a bot.
- ***Random*** [55]: A P2P botnet monitoring algorithm which generates spoof keys uniformly at random. The *discovery ratio* of DUSTBot is expected to be the same as *P2P Zeus*.

4.4.2.2.2 Metrics. Again, the success of anti-crawling countermeasures is measured by the *discovery ratio*, which has been stated in the former experiments.

4.4.2.2.3 Experimental Setup. The size of returned subset *S*_*return*_ is also restricted to *M*/5 in the following experiments. We compare the effectiveness of the proposed anti-crawling countermeasure to the existing countermeasures we stated in Section 3.2.3 by crawling a random bot in the constructed botnet with *ZeusMilker* and *Random*. The crawled bot returns peers with different anti-crawling strategies when it receives a peer list request. Overall, 8 experiments are carried out. 2000 different bots are crawled with different crawling algorithms, and the results are averaged. The performance of *ZeusMilker* is expected to be worse than *Random* because of the additional randomness provided by the latest Ethereum hash.

**Fig 11:**
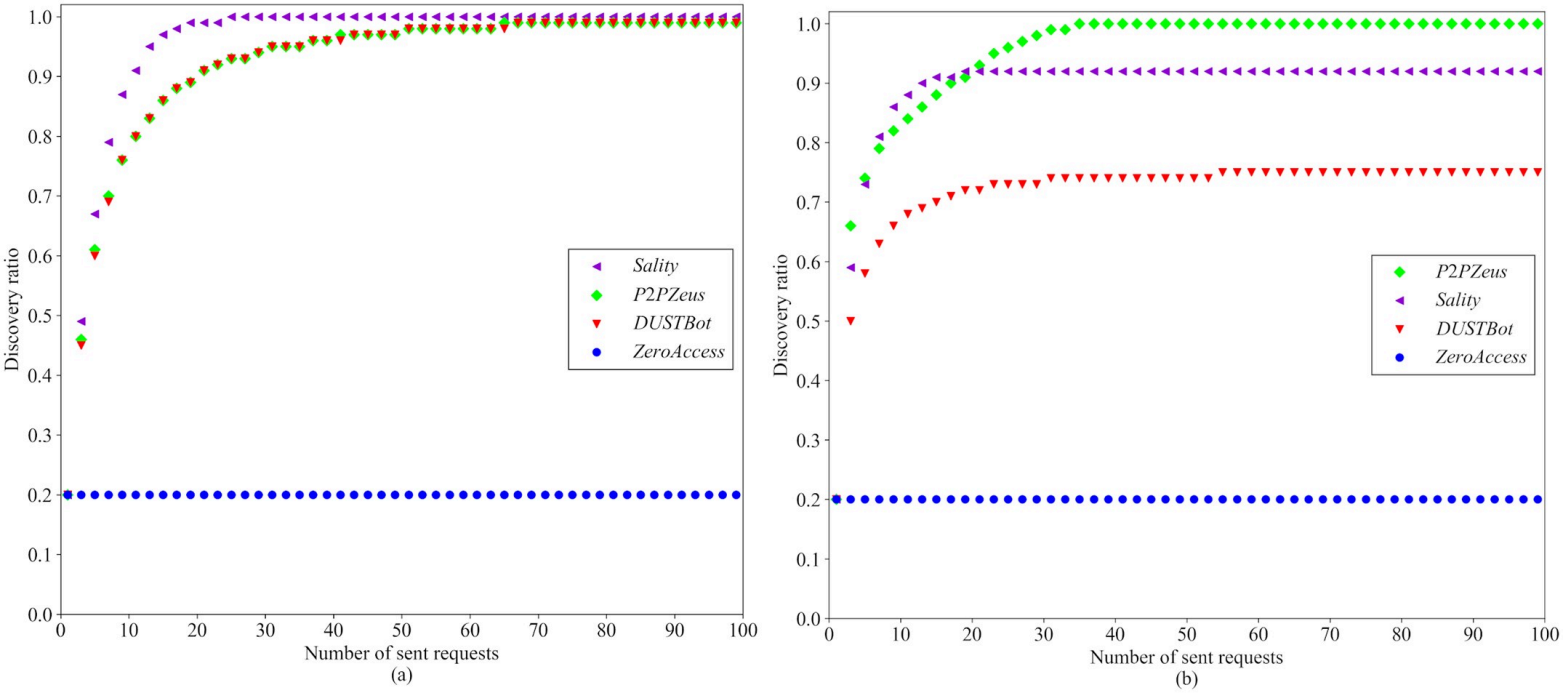
Performance of *Random* (a) and *ZeusMilker* (b) on different anti-crawling strategies (*S*_*return*_ = 4, *M* = 20).

4.4.2.2.4 Results. The performance of *Random* and *ZeusMilker* on a single peer list of different anti-crawling strategies is shown in Fig 11(a) and 11(b).

For *ZeroAccess*, the *discovery ratio* of different crawling algorithms would be precisely 0.2 since a *ZeroAccess* bot always returns first *M*/5 peers in its peer list, and we assume that all the peer list does not change while crawling. Crawling strategies is ineffective to *ZeroAccess*. However, the peer list of a *ZeroAccess* bot is sorted by *most-recently* responsive peers. After the active peers are discovered, a *ZeroAccess* botnet is effectively taken down by defenders.

Fig 11(a) indicates that *Random* is capable of retrieving all peers in the peer list of a bot after a large number of requests except for *ZeroAccess*. DUSTBot and *P2P Zeus* perform better than *Sality* against *Random*. The performance of DUSTBot and *P2P Zeus* against *Random* is similar since the effectiveness of *Random* is not influenced by bits flipping.

The performance of different anti-crawling strategies indicates that DUSTBot performs best with a maximum *discovery ratio* of about 75% after 100 requests, as shown in Fig 11(b). *ZeusMilker* is not capable of discovering all the peers in the peer list of a *Sality* bot since *ZeusMilker* stops crawling when no new key pairs are added to a milking set. DUSTBot performs better than other anti-crawling strategies since additional randomness is added at the side of the queried of a peer list request through the latest Ethereum block hash. It is hard for the requesting node to spoof keys towards a single DUSTBot strategically. The returned subset is biased to a specific key generated by the queried node.

#### 4.4.3 Effectiveness against routing table poisoning attack

The proposed peer list exchange algorithm is also designed to prevent routing table poisoning attack. Each element in the received IP list *L*_*temp*_ has an equal likelihood to be added into the peer list of the requesting node because of the randomness of the latest Ethereum block hash. We will evaluate the effectiveness of the proposed peer list exchange algorithm against the routing table poisoning attack in this subsection.

4.4.3.1 Experimental Setup. To measure the effectiveness of this strategy, we set up an experiment as follows:

**Step 1**: Randomly select a bot *C* whose peer list size is *M* from the botnet constructed in Section 4.1. We assume that 5/*M* of the peers in its peer list are malicious, which intend to inject nodes into the peer list of *C*. And the value of *N*_*need*_ in Algorithm 1 always equals to *M*.
**Step 2**: *C* requests all the peers in its peer list for peers and filters peers to be added utilizing Algorithm 1 for 30000 times. During all the filtering procedures, *C* retrieves the Ethereum block hashes from height 6480001 to height 6510000 as the nonce.
**Step 3**: Collect the results in **Step 2**.
**Step 4**: For each iteration, calculate the fractions that the IP addresses to be added into the peer list of *C* are from malicious peers. Then, the average the results and the standard deviation of the fractions are calculated.

4.4.3.2 Results. According to the results we observed in the evaluation, the average of the fractions is 19.57%, and the standard deviation of the fractions is 0.077, which indicates that each candidate in the received IP list has the equal likelihood to be added into the peer list of the requesting node. The attempt of routing table poisoning attack towards a single DUSTBot would be inefficient because of the additional randomness provided by the latest Ethereum block hash.

### 4.5 DUSTBot stealthiness evaluation

**Fig 12:**
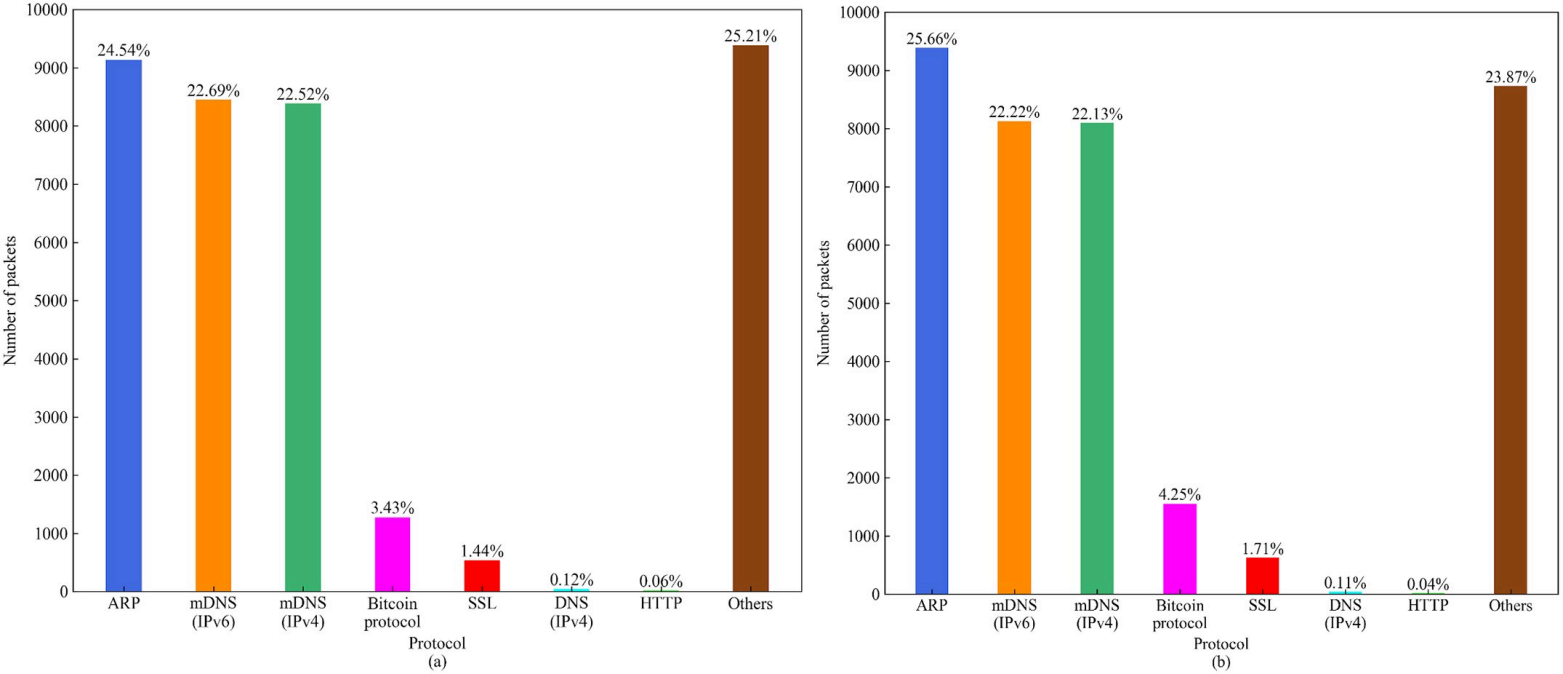
The network traffic of (a) running the Bitcoin Core client and (b) DUSTBot in the same network environment within 15 minutes.

We will evaluate the stealthiness of the proposed botnet through a network traffic analysis in this subsection. To study if DUSTBot is indistinguishable from a genuine Bitcoin user, we individually run the Bitcoin Core Client and DUSTBot on the same computer within the same time interval and monitor the network traffic. The result is shown in Fig 12(a) and 12(b). The number and fraction of the packets of a single DUSTBot (Fig 12(b)) are similar to a genuine Bitcoin user (Fig 12(a)). Hence, the traffic of a single DUSTBot is indistinguishable from that of any other genuine Bitcoin node. The time interval of this experiment is 15 minutes. As throughput, 572 bytes per second is inconspicuous. The result demonstrates the stealthiness of DUSTBot.

## 5. Countermeasures

### 5.1 Countermeasures for the hosts

A DUSTBot listens to port 8333 and 18333, the default ports of the Bitcoin protocol while it is running on an infected device. The communication is easily blocked by filtering network traffic if there is no application based on the Bitcoin protocol on the infected device since DUSTBot disguises itself as a genuine Bitcoin node. However, other network services which rely on the same port would be unavailable if network traffic of port 8333 and 18333 is filtered. Therefore, traffic filtering can be a temporary countermeasure.

### 5.2 Countermeasures for the Bitcoin network

#### 5.2.1 Blocking communication of DUSTBot

Theoretically, the proposed botnet will be shut down if the Bitcoin address that belongs to the botmaster is blacklisted. One possible strategy against DUSTBot is blacklisting the Bitcoin address that belongs to the botmaster with the agreement of the development team of Bitcoin Core since nearly 96% of genuine Bitcoin clients are Bitcoin Core [57].

#### 5.2.2 Tracing the botmaster

Legitimate Bitcoin transactions are broadcast in the Bitcoin P2P network. Therefore, the IP address of the botmaster is exposed to the Bitcoin node to which the transaction data is first sent. Cooperating with the whole Bitcoin network, the source of a Bitcoin transaction could be traced if the Bitcoin address is marked as malicious.

However, the two methods are impractical. The users of Bitcoin would primarily resist such attempts as it would be a behavior that runs against the Bitcoin ethos [58]. Any form of regulation would more or less violate the libertarianism, which is the ideology that Bitcoin persisted. The development team of Bitcoin Core would also refuse to add a blacklist mechanism.

### 5.3 Countermeasures for ISPs

Although tracing and blocking the botmaster is impractical in hosts or the Bitcoin network, transaction data could be captured by SDN-based detection points deployed at ISP level [59] when it is first sent from a host controlled by the botmaster. Then the botmaster could be traced by traditional countermeasures towards botnet C&C servers. However, it is still a challenge for defenders to trace the botmaster by cooperating with so many ISPs around the world since the Bitcoin nodes are distributed globally.

## 6. Discussion

### 6.1 Economic cost of botnet maintenance in the Bitcoin network

At a minimum, it usually costs about 50 cents (USD) per transaction. Assume the botmaster issues a command per 30 minutes, 48 commands are issued every day. Thus the downstream cost is $24.0 every day, $720.0 every month. On the other hand, upstream data is sent back to the botmaster via the Bitcoin testnet. Testnet Bitcoin is free and public to get on some third-party websites. Capturing sensor bots would not lead to money loss to the botmaster. Besides, communication data is broadcast in the self-constructed P2P network. However, the profit of successful botnet is typically hundreds of thousands of dollars per month [60]. Compare to the profits of popular botnets for rent, the cost of the proposed botnet is trivial.

### 6.2 Robustness of the C&C channel of DUSTBot

From what we have discussed above and other studies, there is no easy solutions towards such blockchain “pollution”, dubbed by Forbes magazine [61].

In addition, the network traffic of DUSTBot is indistinguishable from the traffic of a genuine Bitcoin node. Defenders are more possible to distinguish the network traffic generated by bots if they have successfully reverse-engineered a bot. This complicates the analysis toward the proposed botnet. The behavior of a sensor bot is similar to a regular bot, and the types of other bots are not saved in peer lists. This provides a further challenge to distinguish a sensor bot. If the fraction of sensor bots is lower than a threshold, the botmaster can easily upgrade regular bot to sensor bot via the downstream channel, which is hard to block.

The peer list management mechanism, i.e., the peer list exchange algorithm, blacklist mechanism, and segment restriction presented in this paper is effective against DDoS attack and routing table poisoning attack. This provides more challenges for defenders to analyze, monitor, and disrupt the proposed botnet.

## 7. Conclusion and future work

As a preeminent threat to cyberspace, botnets have always been the focus of cybersecurity research. Current solutions to apply blockchain technology to build infrastructure for botnets suffer from high economic cost, single point of failure, and limited scalability. In this paper, we present DUSTBot, a novel P2P botnet model in which C&C communication utilizes the Bitcoin network. Compare to similar works, the C&C channel of DUSTBot is covert, duplex, and low-cost. Besides, the peer list management mechanism we proposed in this paper is effective against routing table poisoning attack and existing botnet crawling algorithms. It is hard for defenders to prevent the botmaster from sending transactions to the Bitcoin main network. Also, it is hard to prevent bots from retrieving commands from the Bitcoin main network. The results demonstrate the feasibility, performance, stealthiness, and robustness of DUSTBot. We are going to make some improvements to our work as follows:

1. Switch C&C channel to other low-cost cryptocurrencies or deploy the C&C communication on multiple cryptocurrencies, which reduces cost and expands the bandwidth of the C&C channel.
2. Try tracing the transaction in the blockchain network of popular cryptocurrencies, to track the botmaster of similar botnets.
3. Try detecting illegitimate network traffic of popular blockchain protocols in hosts with machine learning algorithms against similar botnet models.

## Supporting information

S1 Appendix Methods and experimental data. Detailed descriptions of methods, computational processes, and experimental data. Includes the Bitcoin testnet addresses we used in Section 4.2.
(DOCX)
