# Supplementary material for: DUSTBot: A duplex and stealthy P2P-based botnet in the Bitcoin network
Source: PLoS One. 2019 Dec 20;14(12):e0226594. doi: 10.1371/journal.pone.0226594 (PMC6924649; doi:10.1371/journal.pone.0226594)
Supplement: S1 Appendix — Detailed descriptions of methods, computational processes, and experimental data. Includes the Bitcoin testnet addresses we used in Section 4.2. (DOCX) [file pone.0226594.s001.docx]

**S1 Appendix.**

Methods and experimental data

Supplementary material concerning repeatability

Experiments on the botnet robustness

*1) Metrics: Connected ratio*, the fraction of online peers in the constructed botnet after removing different fractions of sensor bots randomly.

*2) Experiment configurations:* The following configuration is used in the corresponding experiments:

- Parameters for botnet construction:
  - *N_h_* = 20000
  - *M* = 20
  - *p_init_* = 0.25
  - *S_return_* = 4
- Parameters for botnet robustness evaluation:
  - *TTL_up_* ∈ [1, 2]
  - *p_r_* ∈ [0.0, 0.05, 0.1, 0.15, 0.2, 0.25, 0.3, 0.35, 0.4, 0.45, 0.5, 0.55, 0.6, 0.65, 0.7, 0.75, 0.8, 0.85, 0.9, 0.95, 1.0]

In summary, this leads to a total number of 21 * 2 = 42 experiments.

Experiments on the anti-crawling algorithms

*1) Metrics: Discovery ratio*, the fraction of peers discovered during the P2P botnet crawling.

*2) Experiment configurations:* We deployed several crawling algorithms and anti-crawling strategies on the constructed botnet to evaluate the effectiveness of the proposed anti-crawling strategy. The following configuration is used in the corresponding experiments:

- Parameters for botnet construction:
  - *N_h_* = 20000
  - *M* = 20
  - *p_init_* = 0.25
  - *S_return_* = 4
- Deployed anti-crawling strategies:
  - *Sality*: Returns *S_return_* peers in its peer list randomly.
  - *ZeroAccess*: Returns the first *S_return_* elements in its peer list.
  - *P2P Zeus*: Returns *S_return_* peers according to the minimal Kademlia-like XOR distance to the requesting bot’s identifier.
  - DUSTBot: Returns *S_return_* peers according to the minimal Kademlia-like XOR distance to the new identifier with additional randomness, which is generated by the proposed anti-crawling algorithm.
- Deployed crawling algorithms:
  - *BFS* with the following parameters:
    - Number of selected seed peers = 50
    - Maximum number of sent requests = 20000
  - *DFS* with the following parameters:
    - Number of selected seed peers = 50
    - Maximum number of sent requests = 20000
  - *LICA* with the following parameters:
    - Number of selected seed peers = 50
    - Maximum number of sent requests = 20000
    - *r* = 2
    - *w* = 300
    - *t* = 0.1
  - *Random* with the following parameters:
    - Number of selected peers = 2000
    - Maximum number of sent requests = 100
  - *ZeusMilker* with the following parameters:
    - Number of selected peers = 2000
    - Maximum number of sent requests = 100

In summary, this leads to a total number of 20000 * 50 * 3 + 2000 * 100 * 2 = 3400000 experiments.

Experimental data in Section 4.2

The Bitcoin testnet addresses we used is given in this subsection. All the transactions which indicates the feasibility and performance can be accessed by anyone on public blockchain explorers[1–3].

**Table A. The Bitcoin Testnet Addresses Used in Section 4.2.**

| **Role** | **Bitcoin testnet address** |
| --- | --- |
| Botmaster | mh84vzGGA6T5s39BovBJe2dQ3r9KkWQPML |
|  | msV25FyxZJYLSyET8xxCRyYVdbkpp6pv6y |
|  | mgCAkWdsmqKbxBu5Tp95SMxFzXHvLmSdR7 |
| Sensor bot | mgCJA2psRQgjNbX5rRY7998nVF42euissc |
|  | mvcLqrXnpw9iyDhPyFc1iShL21zmoKA9gV |
|  | n4CEVGwr3Sp6saUyrxyBrBDmmmYPXfEatg |
|  | myQM1Hj6xBBBwCCPNtDcQUVMqpwMmfrq2v |
|  | n1hpsTzfTZubrbpd5zJjqEgbJ63i33QULV |
|  | mqkFRhWA7TtsfDwESKZrsEyEDzgD7eDhEw |
|  | mwAgk6heYfzSVpPcgJvD2NwB3rzkaNecEv |
|  | mv5V7rs6VWZL73HuYGf7E3sP9k3nxx21RX |
|  | mqKK3TW3y4zDSEvH3EbjyGibsxa2MsWcAj |
|  | n2aR87aRVKhcKHxinwp6kVpvtDWGoS8dwJ |
|  | mywDdB9hkR5oVBiWjxQSS2Dv6mpr94xLak |
|  | n3VBnsyiPKgWMMk5Sb9ZNUTH57KdSkKUYK |
|  | mnBNRomMTR73kTPrhTgUckDp1s43YtSBLv |
|  | mysR3ESTjZ1G3v4ZdYmfmpjsedck3Rfgdh |
|  | mtYZbWfLKDANi9EXqM6UqttYSfmRjRzd6H |
|  | myX7bjfrXeRY9Ctwf6DFotqzArzN1MaSxL |
|  | msyEpc3eVviA9oT314oeYv2kiNpKR9Em6g |
|  | n3HsN7Pqfxnj967G3vosSJFcDAS9n83XfW |
|  | mk96URVQ5R8nipzP9kRkUw6p9Jn8mMNvuf |
|  | mm6mrC3Mdsdyn4oAyHkpmrG2BMZ3kM2QWp |
|  | myqjuV5Ak5ThXG1m2qcwMt7JYYYDrV4vFg |
|  | mhQ2enqD9aMqENdeik7MVymEpyDgCWKbwS |
|  | mrkTTZFhJFpdstUsckHQCKHFc3oF9vE4T1 |
|  | mpxhVpGWVoSenob6M2yPv82xAY8yYjfn9H |
|  | mtA18mTvMnWAKGtUCppaTx3eUJLg4UjGTo |
|  | mrXwP5j2oKUQzivryQE5EdsnWz2A43vsxf |
|  | n2488BkcMPURZRNuKp3vAueT7zYrRnqfvd |
|  | moiCir3eZwuv5yjxxgafRJbes1nNgFb8LH |

## References

1. Blockchain (2019) Blockchain Explorer.

2. bitaps.com (2019) Bitcoin testnet explorer.

3. BlockCypher (2018) Bitcoin Testnet Block Explorer.
